# Increased Expression of SETDB1 Predicts Poor Prognosis in Multiple Myeloma

**DOI:** 10.1155/2022/3307873

**Published:** 2022-03-23

**Authors:** Jing Xiang, Xiaotong Chen, Mengping Chen, Jian Hou

**Affiliations:** Department of Hematology, Renji Hospital, Shanghai Jiao Tong University School of Medicine, Shanghai, China

## Abstract

Several genes on chromosome 1q21 region predict a high risk of multiple myeloma (MM); however, the underlying molecular pathology remains elusive. Overexpression, amplification, or activation of SET Domain Bifurcated 1 (SETDB1), which is located on 1q21, is closely associated with poor prognosis of many human solid malignancies. In our study, upregulation of SETDB1 might indicate an unfavorable prognosis of MM using bioinformatics analysis from GEO databases and MMRF-CoMMpass. Here, increased SETDB1 expression was observed in the plasma cells from newly diagnosed multiple myeloma patients compared to those from the normal controls. Meanwhile, SETDB1 overexpression was the result of increased copy numbers of SETDB1 gene. In MM patients, the Kaplan-Meier analysis was employed to demonstrate that increased SETDB1 expression was associated with shorter overall survival (OS) and event-free survival (EFS). Besides, we conducted multifactorial cox regression analysis to state that SETDB1 expression was an independent biomarker for OS and EFS. MM patients with higher SETDB1 expression showed higher levels of beta-2 microglobulin (*β*2M), lactate dehydrogenase (LDH), and bone marrow biopsy plasma cells (BMPC) and lower levels of haemoglobin (HGB). Functional enrichment analysis suggested that SETDB1 could promote cell cycle progression in myeloma. Finally, we observed that SETDB1 was distinctly correlated with tumor immunity in MM. SETDB1 expression in myeloma cells was positively correlated with CD56dim natural killer cells but negatively correlated with infiltrating levels of type17 T helper cells, effector memory CD8 T cells, activated dendritic cells, and natural killer T cells from whole bone marrow (WBM) biopsies. Taken together, these results indicated that SETDB1 could be used as a novel biomarker for predicting the prognosis of MM patients.

## 1. Introduction

Multiple myeloma (MM) is a malignant hematologic neoplasm characterized by the infiltration of malignant monoclonal plasma cells in the bone marrow (BM), accompanying with CRAB features consisting of hypercalcemia, renal dysfunction, anemia, and bone disease [1, 2]. MM is a genetically and clinically heterogeneous malignancy caused by multiple genomic events, with the OS duration ranging from a few months to more than a decade [3]. Despite the great advancement in treatment of multiple myeloma due to the advent of several new drugs, most MM patients progress and relapse frequently and remain incurable ultimately. It is for this reason that the discovery of new and promising biomarkers to estimate prognosis and better monitor treatment is essential.

The multitude of genomic events and their concurrency in different patients is the basis for biological heterogeneity in multiple myeloma. It was shown in previous works that cytogenetic abnormalities are regarded as a predictor of prognosis in MM [4]. Gain/amplification of chromosome 1q21 (1q21 gain/amp) is a common and high-risk cytogenetic abnormality found in approximately 40% of newly diagnosed multiple myeloma [5]. The chromosomal aberration of 1q21 locus is directly related with a poorer response and shorter median progression-free survival [6]. Some of the 1q21 genes, such as IL6R [7], ILF2 [8], MCL-1 [9], ADAR1 [10], and CKS1B [11], were investigated and considered potential drivers of MM development and progression. However, many other candidate genes in this region should also be considered for their role in the pathogenesis, progression, or prognosis of myeloma [12].

SETDB1 is mapped to human chromosome 1q21.3, one of seventy-eight amplified or overexpressed 1q21 genes by integrating GISTIC2 and expression data from 246 matched MM samples [8]. Also, systems medicine dissection of chr1q-amp identified 103 candidate genes, including SETDB1, as adverse prognostic biomarkers combined genomic, epigenomic, and transcriptomic data with genetic variables in MM [13]. Besides, the relationship between SETDB1 expression and its prognostic values in multiple myeloma has not been studied. Great efforts of high-throughput sequencing have revealed that overexpression, amplification, or activation of SETDB1 plays an oncogenic effect in malignant cancers, including hepatocellular carcinoma [14], melanoma [15], and glioblastoma [16]. Even though an exact mechanism of action of SETDB1 in cancer has not been revealed, recent reports have established that SETDB1 is involved in gene silencing [17], methylation of Akt [18], suppression of tumor-intrinsic immunogenicity [19], and so on. Hence, SETDB1 possibly acts as an immunosuppressive epigenetic modulator in multiple cancers. Here, we analyzed multiple GEO databases to determine the prognostic effects of SETDB1 expression on MM and its possible action pathways by integrating gene expression and clinical data. Our study found that high expression of SETDB1 is an adverse prognostic marker in MM.

## 2. Methods

### 2.1. Data Source

Supplemental Table 1 shows the clinical details of the multiple gene expression profiling (GEP) and array comparative genomic hybridization (aCGH) datasets from GEO and MMRF-CoMMpass in this research. GEO databases were retrieved and obtained from the Gene Expression Omnibus (https://www.ncbi.nlm.nih.gov/geo/) in the National Center for Biotechnology Information (NCBI). In this present study, clinical information and gene expression profiling microarray were from GSE39754 [20], GSE5900 [21], GSE2658 [22], GSE124435 [23], GSE31161 [24], GSE9782 [25], GSE24080 [26], GSE136337 [27], and GSE136324 [27]. Copy number analysis was conducted to identify genomic regions that are repeated from aCGH data (GSE33685 [28]and GSE26863 [29]). We also integrated and analyzed datasets from the Multiple Myeloma Research Foundation (MMRF) CoMMpass (Clinical Outcomes in MM to Personal Assessment of Genetic Profile) study, which were generated as part of the Multiple Myeloma Research Foundation (MMRF) Personalized Medicine Initiatives (https://research.themmrf.org and http://www.themmrf.org).

We found five MM samples from GSE24080, which were outlier cases based on MAQC's consensus outlier-voting results and should be excluded from model development.

### 2.2. Gene Copy Number Assay

To investigate whether the aberrant expression of SETDB1 was the result of its copy number variation, we finally analyzed 245 patients from GSE26863 who had both copy number and expression values of SETDB1. We performed circular binary segmentation (CBS) of log2ratio values ordered according to their corresponding probes' chromosomal positions and determined each segment's copy number aberration status [10]. Segments mean log2ratio values are above 0.137 and are tagged as gain of SETDB1 copy numbers. Therefore, we could separate these patients into wild type (WT) and copy number gain groups and then compare SETDB1 expression values between these two different groups.

### 2.3. DEG and Functional Enrichment Analysis

Gene expression profile data were downloaded and processed by R and Bioconductor packages. Based on the quartiles of SETDB1 mRNA expression from GSE136337, MM patients were categorized into SETDB1^high^ and SETDB1^low^ groups, with 106 cases in each group. Then, the “limma” package in R (version 3.6.3) [30] was performed to screen for differentially expressed genes (DEGs). Adjusted *p* value < 0.05 and ∣log2 Fold Change | >0.5 were used as the cut-off criteria. In order to visualize DEGs, volcanic maps and heat maps were drawn using the R package “ggplot2” and “pheatmap.”

Kyoto Encyclopedia of Genes and Genomes (KEGG) pathways, Gene Ontology (GO) terms, and Gene Set Enrichment Analysis (GSEA) were all analyzed by “clusterProfiler” package using DEGs to identify biological states and pathway processes affected by SETDB1 [31]. Gene set enrichment analysis was performed using the Broad Molecular Signatures Database (MSigDB v6.0) set H (hallmark gene sets, 50 gene sets) [32]. Nominal *p* < 0.05 was considered the cut-off criterion.

### 2.4. Tumor-Infiltrating Immune Cell (TIC) Proportions and CIBERSORT Algorithm

Based on the clinical and molecular information from GSE136324 and GSE136337, we selected 324 pre-treatment MM samples that had two different gene expression data. These data were produced from whole bone marrow (WBM) biopsies and CD138 +-selected myeloma plasma cells using the Affymetrix Human Genome U133 Plus 2.0 Array (Thermo Fisher Scientific) chips. The expression scores of 27 cell types (22-leukocyte signature matrix and five myeloma-specific cell types) [27] per patient were determined using mRNA expression data of myeloma WBM biopsy samples from GSE156326 dataset through the CIBERSORT [33] algorithm in R (version 3.6.3). We classified MM samples into two groups on the basis of the SETDB1 optimal cut-off value of myeloma cells and then performed immunoinfiltration analysis on these corresponding WBM biopsy samples from GSE156326. We performed Mann–Whitney *U* test to explore differences in fractions of immune-related cells between the SETDB1^high^ and SETDB1^low^ groups.

### 2.5. Tumor-Infiltrating Immune Cell (TIC) Level and Single-Sample Gene Set Enrichment Analysis (ssGSEA) Algorithm

Besides the immune cell proportions, the ssGSEA was implemented to calculate infiltration degrees of 28 immune cell subtypes in above 324 MM samples using the genome variation analysis (GSVA) package [34]. Spearman's correlation analysis was performed to confirm the correlation between SETDB1 expression of myeloma cells and immune infiltration level. According to Jia et al. [35], 12 of 28 tumor-infiltrating immune cells carry out antitumor immunity, like activated CD4 T cells, activated CD8 T cells, central memory CD4 T cells, central memory CD8 T cells, effector memory CD4 T cells, effector memory CD8 T cells, type 1 T helper cells, type 17 T helper cells, activated dendritic cells, CD56bright natural killer cells, natural killer cells, natural killer T cells, and protumor immune cell types included regulatory T cells, type 2 T helper cells, CD56dim natural killer cells, immature dendritic cells, macrophage, MDSCs, neutrophils, and plasmacytoid dendritic cells.

### 2.6. Survival Analysis

We downloaded clinical and prognostic information from GSE24080 and then performed the Kaplan–Meier (K-M) survival analysis to assess the prognostic role of SETDB1 in myeloma patients. We also performed the K-M survival analysis on GSE9782 to verify SETDB1 expression was indeed related to prognosis. Univariate and multivariate cox regression models were used to analyze the correlation of SETDB1 expression with OS and EFS in myeloma patients from GSE24080. Besides, GSE136337 and MMRF-CoMMpass data were used for validation. The MM samples were assigned into high and low expression groups based on the optimal cut-off value of SETDB1 expression, which was determined by the ‘surv_cutpoint' algorithm in the R package “survminer” in these datasets.

### 2.7. Statistical Analysis

When the data were normally distributed, two-tailed unpaired *t*-test and one-way analysis of variance (ANOVA) test were used to compare the mean values of the two and three groups, respectively. The Chi-square test and Wilcoxon test were performed to compare clinical and pathological features between the SETDB1^high^ and SETDB1^low^ groups. Chi-square test was performed for comparison of categorical data, while Wilcoxon test was used for comparison of numerical data. Correlations between the two variables were estimated by Spearman's correlation. The GraphPad Prism 8 or R software (version 3.6.3) were utilized to conduct all the statistical analyses in this paper. *p* < 0.05 was considered statistically significant.

## 3. Results

### 3.1. SETDB1 Was Overexpressed or Amplified in MM Patients

There is no clear recognition of the pivotal driver oncogenes in the 1q21 amplicon until now and more 1q21 cancer-relevant genes need to be identified as potential therapeutic targets. Marchesini et al. [8] found seventy-eight 1q21 genes either amplified or overexpressed by integrating 246 matched MM samples. Also, 103 candidate genes, located in chromosome 1q, were identified as prominent drivers of chr1q-amp multiple myeloma [13]. As a result, twenty-eight genes were both detected, including MCL1, ILF2, ADAR, CKS1B, and SETDB1 (supplemental Figure 1A). Then, we searched for the functions of these genes and whether they had been studied in myeloma. Few studies have been done on the relationship between SETDB1 and multiple myeloma, and we supposed that SETDB1 could act as a novel poor prognostic biomarker.

**Figure 1:**
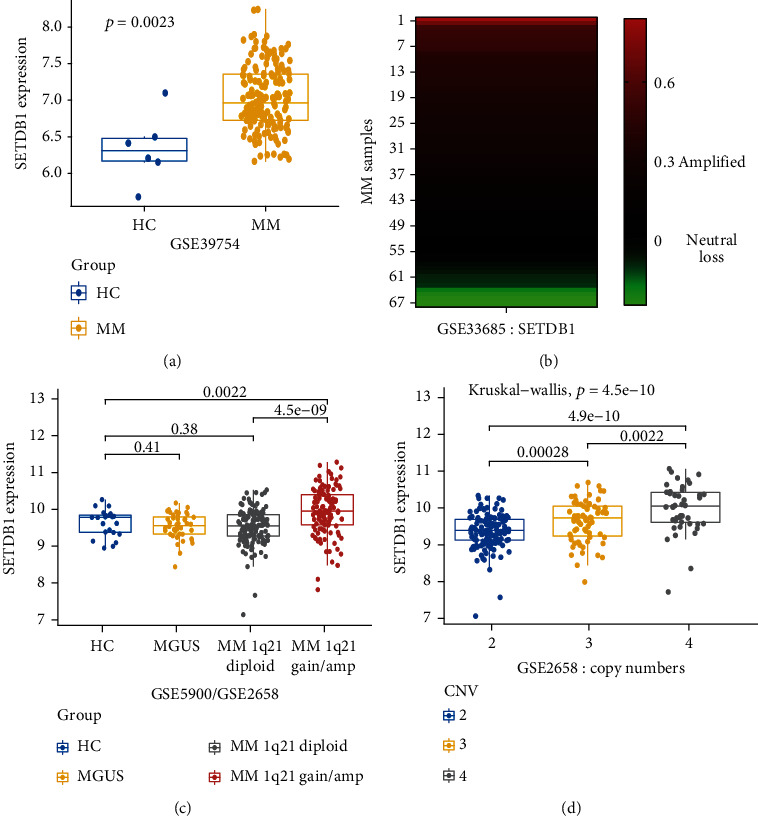
Different expression patterns of SETDB1 in multiple myeloma. (a) SETDB1 expression of plasma cells from the healthy control (*n* = 6) and newly diagnosed myeloma patients (*n* = 170) in GSE39754 database (*p* < 0.01, Wilcoxon test). (b) Heatmap was illustrating SETDB1 copy number variation in 67 NDMM samples. The right *y*-axis represents MM samples and the scale color bar indicates normalized log ratios obtained from LogRatio in raw data, calculated by Log10(rProcessedsignal/gProcessed signal). Patient with Log10 copy number ratio ≥ 0.15 were considered as gain and ratio ≥ 0.3 were considered amplification. (c) Comparison of SETDB1 mRNA levels at HC, MGUS, 1q21 diploid MM patients, and 1q21 gain/amplification MM samples. (d) Comparison of SETDB1 expression at different levels of 1q21 copy numbers. The *x*-axis represents different groups, and the *y*-axis represents gene expression (*p* < 0.01, ANOVA test). SETDB1 gene expression was measured as log2.

Firstly, we calculated the mRNA expression of SETDB1 of plasma cells from the healthy controls (HC, *n* = 6) and newly diagnosed myeloma patients (NDMM, *n* = 170) in GSE39754 database. The result showed that there were substantial differences of SETDB1 expression between multiple myeloma cells (MMCs) and normal plasma cells (NPCs) (Figure 1(a),*p* < 0.01, Wilcoxon test). Besides, subsequent analysis of GSE33685, an array-based comparative genomic hybridization (aCGH) data including 67 NDMM samples, demonstrating that amplification of the SETDB1 gene was detected in 39 of 67 (58%) MM patients (Figure 1(b)). There was no statistically significant difference in expression levels among the healthy control (HC, *n* = 12), monoclonal gammopathy of unknown significance (MGUS, *n* = 44), and 1q21 diploid MM patients (*n* = 134); however, compared with the healthy control and 1q21 diploid MM patients, SETDB1 expression in the 1q21 gain/amplification MM samples was significantly increased (*n* = 114) (Figure 1(c), *p* < 0.01, unpaired *t*-test; *p* < 0.01, Wilcoxon test). Notably, expression of SETDB1 elevated with increasing copy numbers of 1q21 in the GSE2658 MM sample set (Figure 1(d), *p* < 0.01, Kruskal-Wallis). We next assessed whether overexpression of SETDB1 was cooccurrent with detecting translocations or copy number changes from MMRF-CoMMpass. The NDMM patients were separated into seven groups based on the cytogenetic abnormalities (1q21 gain/*n* = 119, 17p del/*n* = 17, *t*(8; 14)/*n* = 37, *t*(4; 14)/*n* = 33, *t*(14; 16)/n = 8, *t*(11; 14)/n = 87, and normal karyotype/*n* = 196). Overexpression of SETDB1 was observed only in the MM patients with 1q21 gain or *t*(11; 14) than the normal karyotype group. Meanwhile, SETDB1 expression was increased in 1q21 gain MM patients than all other six groups (supplemental Figure 1B, all *p* < 0.01, unpaired *t*-test).

From GSE124435, this research found SETDB1 expression from circulating clonal plasma cells (CTC) was positively correlated with expression from bone marrow clonal plasma cells (BMPC) (Supplemental Figure 1C, *r* = 0.45, *p* < 0.05). SETDB1 expression of CTC was also increased in relapsed multiple myeloma compared to NDMM patients (supplemental Figure 1D, *p* < 0.05). Thus, the combined analysis of multiple public data sources demonstrated that SETDB1 was remarkably upregulated in MM cases.

### 3.2. Increased SETDB1 Copy Numbers Contributed to the Overexpression of SETDB1

**Figure 2:**
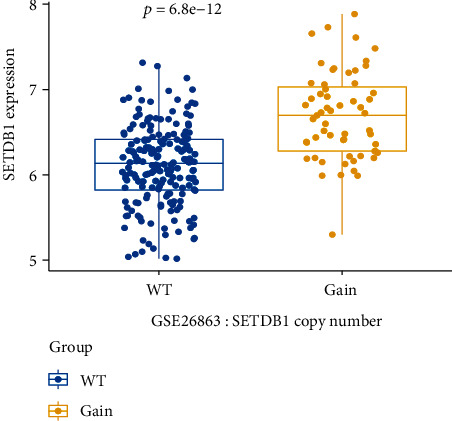
SETDB1 copy number variation and mRNA level. Box plot based on the dataset GSE26863 shows that increased SETDB1 expression was the result of upregulated SETDB1 gene copy numbers (WT, *n* = 186, Gain, *n* = 159, *p* < 0.01, Wilcoxon test). The *x*-axis represents SETDB1 copy number gain or not, and the *y*-axis represents gene expression. SETDB1 gene expression was measured as log2.

As SETDB1 mRNA levels were strikingly upregulated in multiple myeloma, we would like to explore the potential mechanisms underlying this phenomenon. Amplification of 1q21 locus is observed in 40% of NDMM patients and SETDB1 gene is located within chr1q21. We then examined the relationship between DNA copy number changes and SETDB1 gene expression in GSE26863 dataset, which contained 304 GEP and 254 aCGH of purified myeloma cells from NDMM patients. 245 samples with both Affymetrix gene expression microarray and the Agilent CGH microarray data were included in the follow-up analysis. Segment mean log2ratio values are above 0.137 which are tagged as gain of SETDB1 copy numbers. Therefore, we separated these patients into wild type (WT) and copy number gain groups. Then, we compared SETDB1 expression values between the two groups. Notably, an increase in SETDB1 gene copy numbers was linked to overexpression of SETDB1 in the GSE26863 sample set (Figure 2, *p* < 0.01, Wilcoxon test). The finding suggested that increased SETDB1 copy numbers greatly contributed to the abnormal expression of SETDB1.

### 3.3. Increased SETDB1 Expression Was Linked to Disease Relapse and Worse Clinical Outcomes in Different Treatment Protocols

**Figure 3:**
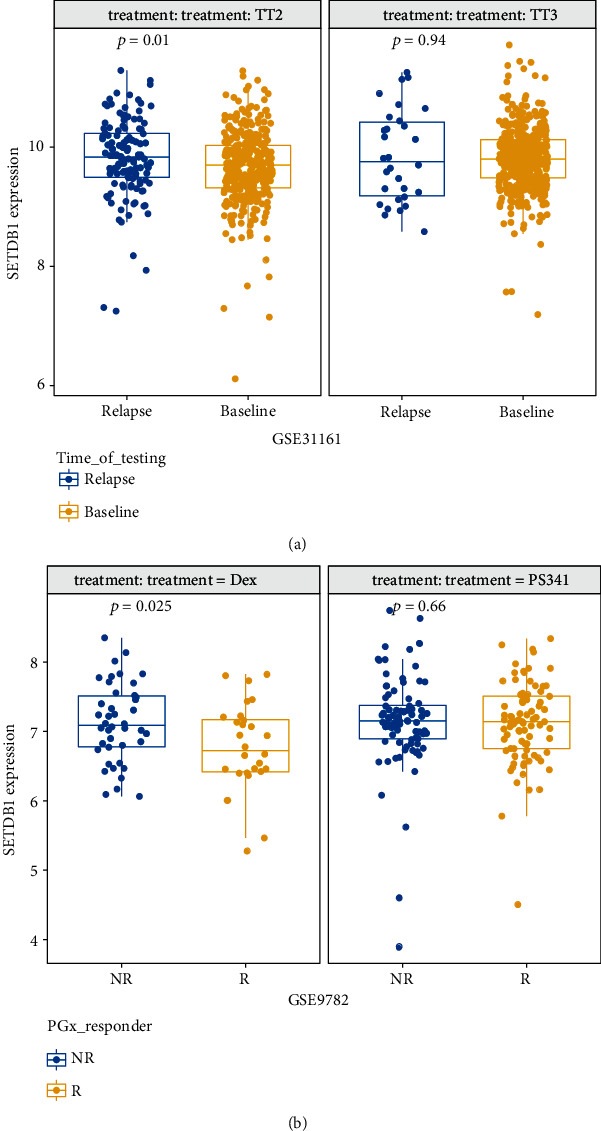
SETDB1 expression in different disease stages and treatment response. (a) SETDB1 expression was increased in the relapsed group (*n* = 347) in comparison with the baseline group (*n* = 127) in the TT2 group from GSE31161 (*p* < 0.05, Wilcoxon test), but there was no difference in the TT3 group. (b) SETDB1 expression was different in MM patients with the response (*n* = 28) and no response (*n* = 42) in the DEX treatment group from GSE9782 (*p* < 0.05, Wilcoxon test), and there was no difference in the PS341 treatment group. The *x*-axis represents different groups, and the *y*-axis represents gene expression. SETDB1 gene expression was measured as log2.

Since SETDB1 was upregulated in NDMM patients, we want to explore whether SETDB1 expression would be altered after treatment and different treatment protocols would change the prognosis in high expression patients. To figure out this problem, we analyzed SETDB1 mRNA level variations under different situations in two public datasets. In GSE31161 sample set, increased SETDB1 expression was observed in relapsed MM patients when compared to baseline expression in the TT2 group; however, there was no difference in the TT3 group (Figure 3(a), TT2 *p* < 0.05; TT3 *p* = 0.94, Wilcoxon test). The main difference between TT2 and TT3 was that TT2 contained thalidomide and TT3 contained bortezomib. Similarly, according to the last response of MM patients from GSE9782, we considered patients whose clinical outcome superior to partial response (PR) to be responders; meanwhile, progressive disease (PD) and relapse represented nonresponse. SETDB1 expression was slightly elevated in the response group in comparison to the no-response group when treatment was dexamethasone (Dex), but there was no difference in the bortezomib (PS341) treatment group (Figure 3(b), *p* < 0.05, *p* = 0.66, respectively, Wilcoxon test). To sum up, SETDB1 high expression was related to disease relapse and worse clinical outcomes in different treatment protocols.

### 3.4. High SETDB1 Expression Predicted Poor Survival in MM Patients

**Figure 4:**
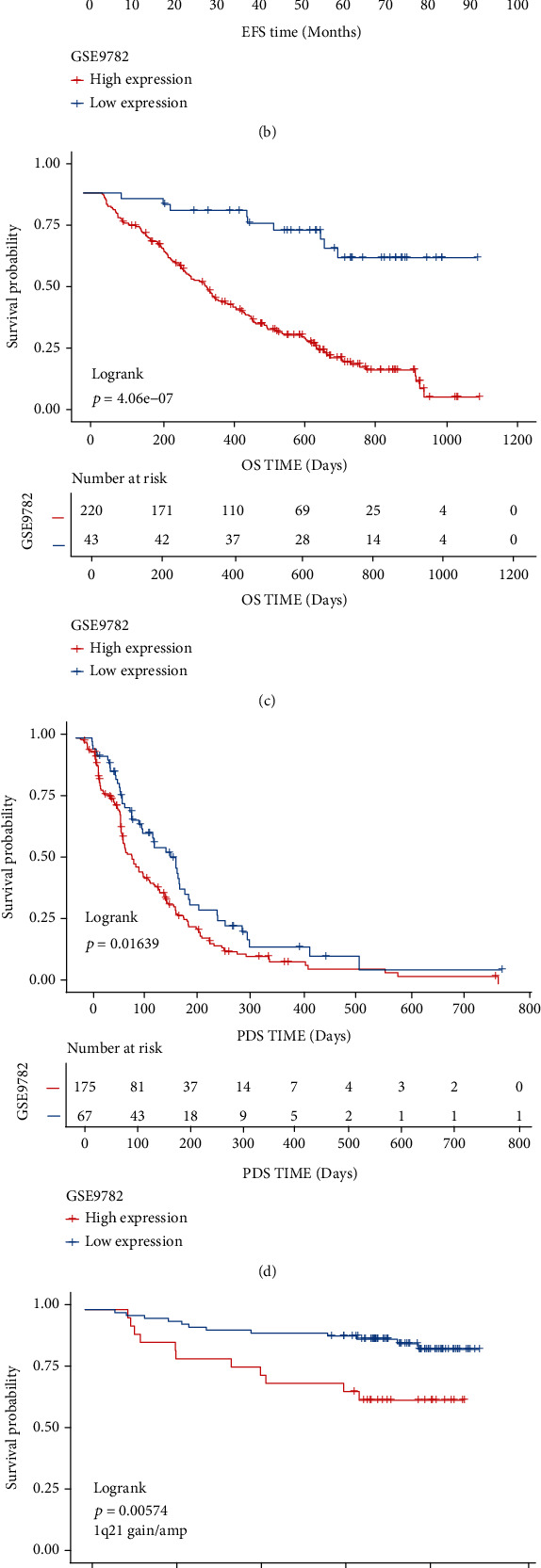
Survival analysis of the SETDB1^high^ and SETDB1^low^ groups. (a and b) The Kaplan–Meier survival curves show that high SETDB1 expression predicts poor OS time and EFS time in MM sample from GSE24080 according to the SETDB1 optimal cut-off value (*p* < 0.01 and *p* < 0.01, respectively, log-rank test). (c and d) The Kaplan–Meier survival curves show that high SETDB1 expression predicts poor OS time and EFS time in the MM sample from GSE9782 according to the SETDB1 optimal cut-off value (*p* < 0.01 and *p* < 0.05, respectively, log-rank test). (e and f) The Kaplan-Meier analysis on the overall survival of MM patients from GSE2658 with and without 1q21 gain/amplification group (*p* < 0.01 and *p* < 0.01, respectively, log-rank test). The *x*-axis represents time, and the *y*-axis represents survival probability.

To explore the predictive value of SETDB1, we divided 554 MM patients from GSE24080 into two different groups on the ground of the optimal cut-off value of SETDB1 expression. The SETDB1^high^ group was strongly related to adverse survival in MM, while the SETDB1^low^ group showed a better survival (Figure 4(a), OS, *p* < 0.01; Figure 4(b), EFS, *p* < 0.01, log-rank test). Similarly, shorter overall survival and progress-free survival (PFS) were observed in the SETDB1 high expression group than the low expression group from GSE9782 (Figure 4(C), OS, *p* < 0.01; Figure 4(d), PFS, *p* < 0.05, log-rank test). Since SETDB1 expression may correlate with 1q21 gain/amp, we analyzed the prognosis value of SETDB1 in two groups with and without 1q21 gain/amp from GSE2658. The findings proved that high SETDB1 expression patients presented with worse survival (Figure 4(e), *p* < 0.01; Figure 4(f), *p* < 0.01, log-rank test). Multivariate cox regression analysis was also performed to assess the predictive value of SETDB1 (Table 1), and these findings indicated that high SETDB1 expression in multiple myeloma was an independent unfavorable prognostic factor for OS and EFS (*p* < 0.05). In conclusion, these data suggested high expression of SETDB1 was an adverse prognosis factor in multiple myeloma.

HR: hazard ratio; CI: confidence interval; CREAT: creatinine, mg/dl; LDH: lactate dehydrogenase, U/l; ALB: albumin, g/l; HGB: haemoglobin, g/dl; MRI: numbers of magnetic resonance imaging- (MRI-) defined focal lesions (the skull, spine, and pelvis); Cyto.abn: an indicator of the detection of cytogenetic abnormalities.

### 3.5. SETDB1 Expression Was Related to Clinical Characteristics

Since SETDB1 was aberrantly upregulated in MM samples and identified as an independent unfavorable prognostic factor, we subsequently examined clinical significance of SETDB1 expression in MM samples from GSE24080 dataset. We separated these patients into high and low groups depending on the median SETDB1 expressions and then tested them in predicting the distribution of clinicopathological features. By utilizing multiple clinical characteristics, we found different distributions between the two subgroups in GSE24080 MM patients (Table 2). Between the two groups, SETDB1 expression was linked with clinical parameters like myeloma isotype and cytogenetic abnormalities (*p* < 0.05, Chi-squared test). Additionally, MM patients with high SETDB1 expression were more likely to obtain higher *β*2M (beta-2 microglobulin), LDH (lactate dehydrogenase), BMPC (bone marrow biopsy plasma cells), and lower HGB (haemoglobin), which were all essential biomarkers in MM prognosis (all *p* < 0.05, Wilcoxon test).

Age: age at registration (years); B2M: beta-2 microglobulin, mg/l; CRP: c-reactive protein, mg/l; CREAT: creatinine, mg/dl; LDH: lactate dehydrogenase, U/l; ALB: albumin, 35 g/l; HGB: haemoglobin, g/dl; ASPC: aspirate plasma cells (%); BMPC: bone marrow biopsy plasma cells (%); MRI: numbers of magnetic resonance imaging- (MRI-) defined focal lesions (the skull, spine, and pelvis); Cytogenetic abnormality: an indicator of the detection of cytogenetic abnormalities; no: number of patients. ^#^Chi-squared test, ^†^Wilcoxon test.

### 3.6. SETDB1 May Exert an Important Role in the Cell-Cycle Progression of MM

**Figure 5:**
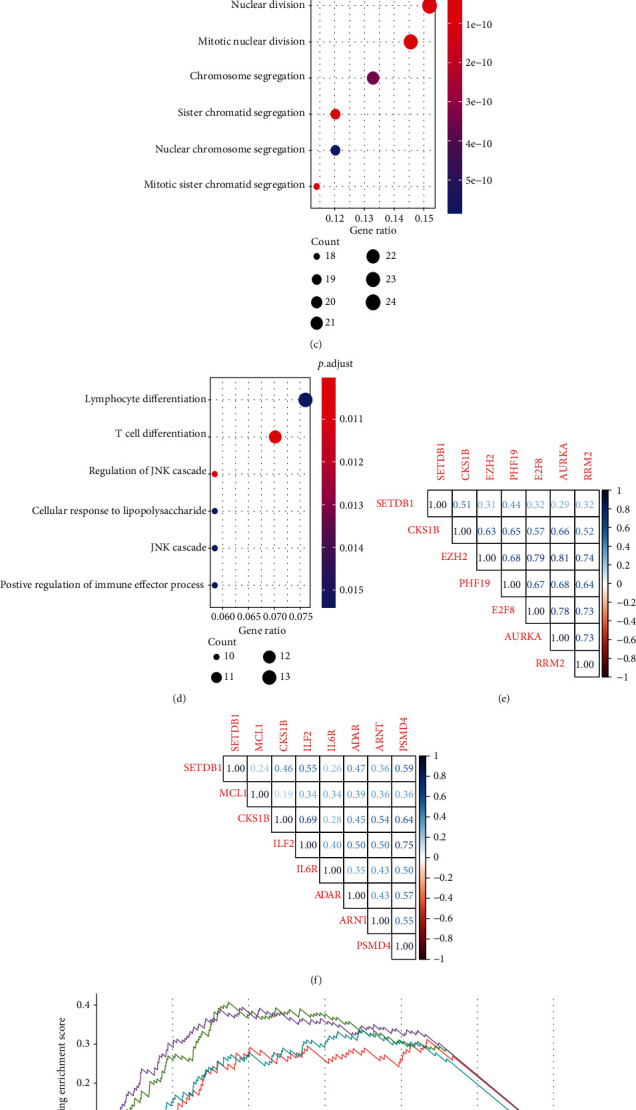
Different expression genes (DEGs) and functional enrichment analysis. (a) Volcano plot of the DEG expression between SETDB1high and SETDB1low from GSE136337. The *y*-axis displays the -log10 adjusted *p* value for each gene, while the *x*-axis displays the log2 fold change for that gene relative to SETDB1 expression. (b) KEGG results for upregulated genes. (c) GO results for upregulated genes. (d) GO results for downregulated genes. (e) The correlation coefficient between SETDB1 and CKS1B, EZH2, PHF19, E2F8, AURKA, and RRM2. (f) The correlation coefficient between SETDB1 and MCL1, CKS1B, ILF2, IL6R, ADAR, ARNT, and PSMD4. (g) GSEA analysis shows that many genes positively correlated with SETDB1 are involved in four different pathways.

To further determine the molecular mechanisms underlying protumorigenic effect of SETDB1 in myeloma cells, we firstly performed differential gene expression analysis between the SETDB1high (*N* = 106) and SETDB1low (*N* = 106) groups from GSE136337 [27]. All told there were 364 differential genes identified in the two groups by bioinformatics analysis, among which 170 genes were upregulated and 194 genes significantly downregulated in the SETDB1high group (∣log2FC | >0.5, adjusted *p* < 0.05) (Figure 5(a)). We then utilized DEGs to analyze the KEGG pathways and GO terms to illuminate the biorole of SETDB1. In the result of KEGG and GO analysis, SETDB1 is highly associated with cell cycle, DNA replication, and mitotic nuclear division (Figures 5(b)–5(d), adjusted *p* < 0.05). Furthermore, we selected 3286 genes that were most relevant to SETDB1 (Spearman's correlation value > 0.3 or < −0.3, *p* < 0.05) in MM samples from GSE136337. SETDB1 was a highly positive correlation with CKS1B, EZH2, PHF19, E2F8, AURKA, and RRM2, which are involved in the cell cycle (Figure 5(e)). SETDB1 high expression myeloma cells exhibited increased expression of those cell cycle-related genes (supplemental Figure 2A). Also, we found SETDB1 was positively correlated with common pathogenic 1q21 genes with *r* values from 0.24 to 0.59 (Figure 5(f)). Then, we performed gene set enrichment analysis (GSEA) using the MSigDB hallmark gene sets, which revealed that genes positively correlated with SETDB1 were enriched in “HALLMARK_G2M_CHECKPOINT”, “HALLMARK_MYC_TARGETS_V1”, “HALLMARK_DNA_REPAIR”, and “HALLMARK_E2F_TARGETS” (Figure 5(g), adjusted *p* < 0.05). In addition, we found proliferation index was elevated in MM patients of high SETDB1 expression from MMRF-CoMMpass (supplemental Figure 2B). Thus, we supposed that SETDB1 might act as an oncogene, which exerts an important role in the cell cycle progression of MM.

### 3.7. SETDB1 Was Correlated with Tumor-Infiltrating Cells in MM

**Figure 6:**
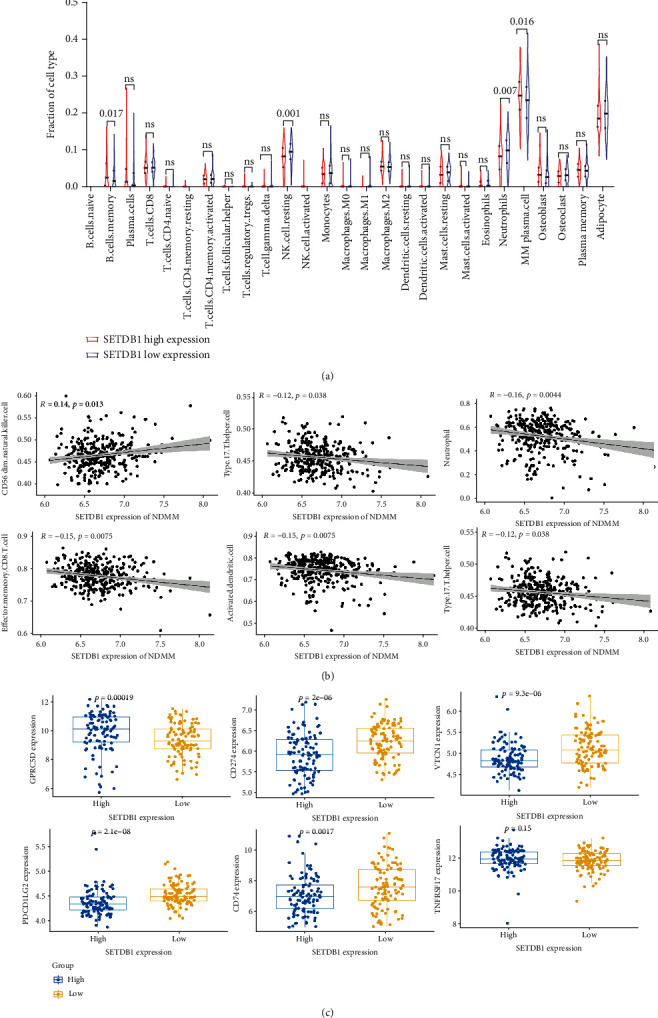
The association of SETDB1 expression of myeloma cells and immune cell infiltration levels. (a) The Violin plot showed the proportion of 27 kinds of cell types in the high (*n* = 104) and low (*n* = 222) SETDB1 expression groups in MM samples. (b) The Scatter plot showed the associations between SETDB1 mRNA levels and infiltrations of immune cells in multiple myeloma WBM biopsies. (c) The Boxplot showed the distribution of immune checkpoint markers between the high- and low-expression of SETDB1 in myeloma samples. *p* < 0.05 as statistically significant. GPRC5D: G protein-coupled receptor class C group 5 member D; CD274: Programmed Cell Death 1 Ligand 1; VTCN1: V-Set Domain-Containing T Cell Activation Inhibitor 1; PDCD1LG2: Programmed Cell Death 1 Ligand 2; TNFRSF17: TNF Receptor Superfamily Member 17.

Previous researches have illustrated a point that TICs (Tumor-infiltrating immune cells) influence disease progression and clinical outcomes in MM [36].  Figure 5(d) shows that high SETDB1 expression might influence cell differentiation and regulation of the immune effector process. To explore the association between SETDB1 and tumor microenvironment (TME), we utilize the CIBERSORT algorithm to compute the proportions of TICs in MM samples. Then, we implemented the analysis between the expression of SETDB1 and TIC abundance. Based on the optimal cut-off value of SETDB1 expression from GSE136337, we divided these 326 myeloma plasma samples into two groups. We then performed immunoinfiltration analysis on these 326 WBM biopsy samples from GSE136324. We found that SETDB1 high expression myeloma samples exhibited higher percentages of memory B cells and MM plasma cells while lower percentages of resting NK cells and neutrophils (Figure 6(a), all *p* < 0.05, Wilcoxon test). For validation, the ssGSEA algorithm was utilized to investigate the correlation between SETDB1 expression of myeloma cells and immune infiltrating levels (Figure 6(b)). Results further indicated that SETDB1 was positively correlated with CD56dim natural killer cells (*r* = 0.14, *p* < 0.01) but negatively correlated with infiltrating levels of type17 T helper cells (*r* = −0.19, *p* < 0.01), neutrophils (*r* = −0.16, *p* < 0.01), effector memory CD8 T cells (*r* = −0.15, *p* < 0.01), activated dendritic cells (*r* = −0.15, *p* < 0.01), and natural killer T cells (*r* = −0.12, *p* < 0.05). Gabriel et al. [19] verified that SETDB1 amplification in human tumors was involved in immune evasion and resistance to immune checkpoint blockade. We observed that immune checkpoint markers, consisting of PD-L1, VTCN1, and PDCD1LG2, were remarkably downregulated in SETDB1 high expression MM patients (Figure 6(c), all *p* < 0.01, Wilcoxon test). The evidence confirmed that SETDB1 was related to immune infiltrating cells and played a key role in tumor immune escape in multiple myeloma.

#### 3.7.1. Validation Using Independent External Databases

**Figure 7:**
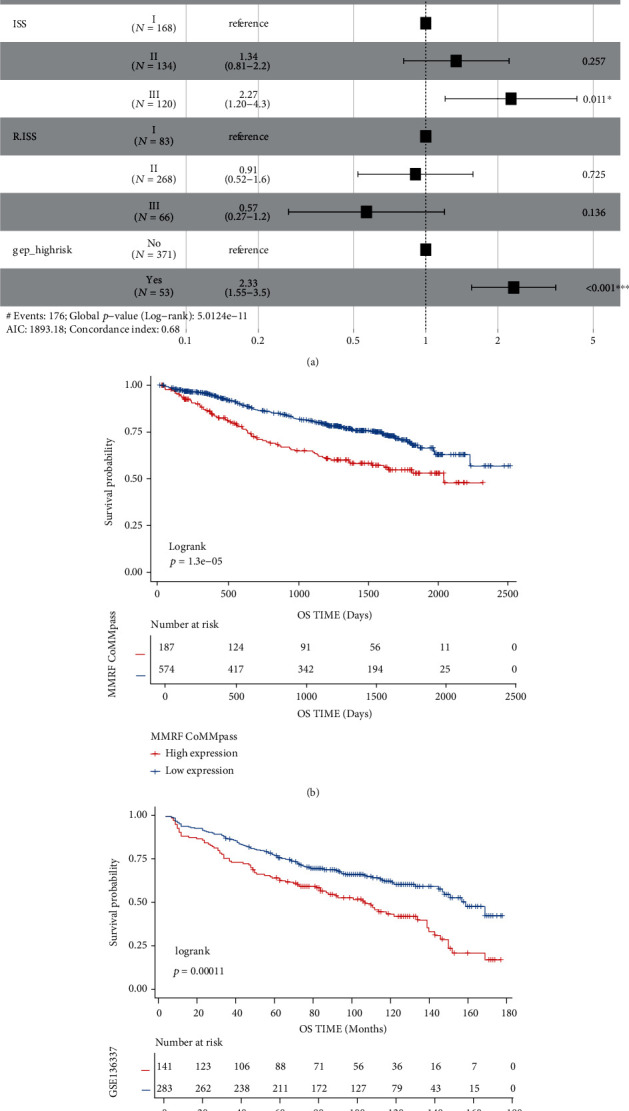
SETDB1 indicated poor prognosis in the other two validation databases. (a) Forest plot of the multivariate cox regression analysis for OS in GSE136337. (b and c) The Kaplan-Meier survival curve for OS in two validation datasets MMRF-CoMMpass and GSE136337 (*p* < 0.01 and *p* < 0.01, respectively).

We made further validation of the reproducibility and accuracy of SETDB1 expression in the prognostic impact of myeloma patients by analyzing two additional independent and large sample datasets, consisting of MMRF-CoMMpass and GSE136337. Multivariate cox analysis was applied to assess that upregulated SETDB1 expression further was an independent unfavorable prognostic biomarker for OS in GSE136337 database (Figure 7(a), HR = 2, *p* < 0.01). Moreover, in these two validation sets, the K-M survival analysis revealed that MM with increased SETDB1 expression was remarkably associated with decreased OS (Figures 7(b) and 7(c), *p* < 0.01 and *p* < 0.01, respectively, log-rank test). Hence, SETDB1 was a potential biological marker to predict the prognosis of MM cases.

## 4. Discussion

Despite significant advances in the treatment of MM patients, drug resistance and recurrent relapses continue to characterize the disease and ultimately lead to death from the disease. Combination therapy and identification of predictive biomarkers are essential to improve treatment outcomes in newly diagnosed and relapsed/refractory MM. Integrating and analyzing multiple independent GEO datasets or MMRF-CoMMpass in this study for differential gene expression analysis, survival analysis, and other bioinformatics analysis methods, we revealed that SETDB1 was significantly overexpressed and amplified in MM patients compared with the healthy controls. Increased SETDB1 gene copy numbers contributed significantly to the overexpression of SETDB1. In addition, high SETDB1 expression was related to the adverse outcome of MM, indicating that it may be a carcinogenic gene and prognostic biomarker.

In the last decade, researchers have intensively investigated the impact of epigenetics on disease. It is well known that epigenetic processes comprising DNA methylation, histone modifications, and noncoding RNAs have an impact on the pathogenesis and progression of MM [37, 38]. Some epigenetic markers have been going into clinical trials in multiple myeloma [39, 40]. SETDB1 is aberrantly expressed in a wide variety of human cancers, facilitating tumor development and drug resistance. Through methylation of p53, SETDB1 regulates the growth of hepatocellular carcinoma cells [41]. It is also confirmed that SETDB1 could bind to the promoter of p21 and silence its expression, promoting the progression of human colorectal cancer [42]. SETDB1 also enhances the protein expression of c-MYC and CCND1 to promote breast cancer cell cycle progression. In turn, c-MYC enhances SETDB1 transcription, indicating a positive regulatory role between SETDB1 and c-MYC [43]. In addition to its primary function as a histone H3K9 methyltransferase, SETDB1 can act on the methylation of other proteins, like maintaining the stability of the p53 protein [41], to play its carcinogenic effect. While the role of SETDB1 in tumors has not been completely delineated, most studies suggest that SETDB1 has prooncogenic potential by regulating key tumor-associated genes. These studies may provide a new therapeutic target for the clinical treatment of human cancers.

In our analysis, SETDB1 expression levels were significantly upregulated in myeloma samples. SETDB1 is one of seventy-eight amplified or overexpressed 1q21 genes by integrating 246 matched MM samples [8]. Consistent with our findings, a frequent amplification (58%) of the SETDB1 gene was observed in MM samples from GSE33685. It is worth noting that increased SETDB1 gene copy numbers greatly promote the paradoxical SETDB1 expression. Moreover, high expression of SETDB1 was related to a striking reduction of EFS, PFS, and OS time in MM samples. Like LDH and ALB, SETDB1 could be regarded as an independent prognostic factor for MM. Notably, we observed that SETDB1 was distinctly correlated with tumor immunity in MM. We discovered that SETDB1 expression of myeloma cell was positively correlated with CD56dim natural killer cells but negatively correlated with infiltrating levels of type17 T helper cells, neutrophils, effector memory CD8 T cells, activated dendritic cells, and natural killer T cells from whole bone marrow (WBM) biopsies. Our data offered support for the possible involvement of SETDB1 in regulating immune infiltration of myeloma tumor environment. Nevertheless, clinical and experimental validation is needed to investigate the functions and pathways of SETDB1 in tumor immune infiltration of MM. Apart from that, we found that SETDB1 may likely have far-reaching effects on the cell cycle, DNA replication, and mitotic nuclear division in our research. Yu et al. found that upregulation of SETDB1 enhanced the transcription of CCND1 and CDK6, thereby promoting proliferation of colorectal cancer cells [44]. Shang et al. observed that high SETDB1 expression may accelerate cell proliferation through the ERG-CCND1/MMP9 axis, which was also associated with adverse prognosis in gastric cancer [45]. Hence, it has been demonstrated that SETDB1 may regulate the cell cycle genes leading to disease progression in multiple myeloma.

Our study firstly revealed the prognostic function of SETDB1 expression in multiple myeloma. However, the role of SETDB1 gene has not been thoroughly investigated. And more research and experiments need to be done in the future to investigate the mechanism of SETDB1 involvement in MM tumorigenesis, development, and drug resistance.

## 5. Conclusions

This study firstly provided all-around evidence for the expression of SETDB1 in myeloma and its potential as a biotarget and prognostic predictor of MM. We have demonstrated that increased SETDB1 gene copy numbers contributed significantly to the aberrant SETDB1 expression and high SETDB1 expression was an unfavorable prognostic biomarker in myeloma patients. Notably, we observed that SETDB1 was distinctly correlated with tumor immunity in MM. Furthermore, we found that SETDB1 may exert an important role in the cell-cycle progression of MM. However, the molecular mechanism of SETDB1 in MM has not been fully certified. More research and experiments need to be done to explore the mechanism of SETDB1 participating in MM tumorigenesis, development, and drug resistance in the future.

## Figures and Tables

**Table 1:** Multivariate analysis for EFS and OS in MM patients from GSE24080.

Characteristics	Total	Overall survival			Event-free survival		
		HR	*p* value	95% CI	HR	*p* value	95% CI
SETDB1	*N* = 554	1.384	<0.05	1.052-1.819	1.273	<0.05	1.014-1.597
CREAT	*N* = 551	1.138	<0.01	1.042-1.243	1.193	<0.01	1.099-1.296
LDH	*N* = 554	1.005	<0.01	1.003-1.007	1.004	<0.01	1.002-1.006
ALB	*N* = 554	0.640	<0.01	0.507-0.807	0.775	<0.05	0.633-0.949
MRI	*N* = 517	1.014	<0.01	1.005-1.023	1.008	<0.05	1.000-1.016
Cyto.Abn	*N* = 554	1.951	<0.01	1.416-2.688	1.593	<0.01	1.221-2.078

**Table 2:** The association of SETDB1 expression and clinical characteristics in MM samples from GSE24080.

Characteristics	Total	High expression	Low expression	*p* value
Sex				
Female	*N* = 554	117	103	0.224^#^
Male		160	174	
ISOTYPE				
Light chain	*N* = 536	42	41	<0.05^#^
IgA		80	52	
IgG		144	167	
Other		2	8	
MRI (numbers of magnetic resonance imaging (MRI)-defined focal lesions spine) pelvis))				
≥ 3	*N* = 517	148	153	0.694^#^
<3		110	106	
Cyto.Abn (An indicator of the detection of cytogenetic abnormalities)				
Yes	*N* = 554	114	90	<0.05^#^
No		163	187	
Age (years)(mean(range))	*N* = 554	57.81 (29.7-75.94)	56.61 (24.83-76.5)	0.099^†^
*β*2M (mg/l)(mean(SD))	*N* = 553	5.205 (5.8479)	4.166 (4.6239)	<0.01^†^
CRP (mg/l)(mean(SD))	*N* = 550	11.398 (21.5856)	11.843 (24.5054)	0.746 ^†^
CREAT (mg/dl)(mean(SD)	*N* = 551	1.359 (1.3162)	1.272 (1.2187)	0.315^†^
LDH (U/l)(mean(SD))	*N* = 554	177.69 (68.69)	165.34 (61.346)	<0.05^†^
ALB (g/l)(mean(SD))	*N* = 554	4.015 (0.6326)	4.089 (0.5222)	0.509^†^
HGB ((g/dl)(mean(SD))	*N* = 554	11.070 (1.8093)	11.445 (1.7988)	<0.05^†^
ASPC (%) (mean(SD))	*N* = 526	43.858 (25.2960)	41.235 (23.1060)	0.274^†^
BMPC (%) (mean(SD))	*N* = 539	49.390 (26.723)	43.380 (25.5590)	<0.01^†^

## Data Availability

MMRF-CoMMpass and GEO belong to public databases. The datasets of this report were generated by GEO: https://www.ncbi.nlm.nih.gov/geo/ and MMRF-CoMMpass: https://research.themmrf.org

## Supplementary Materials

Supplementary Materials Supplemental Table 1: the information of public databases analyzed in this study. Supplemental Figure1: SETDB1 expression profile of myeloma cells in different databases. Supplemental Figure 2: the potential impact of SETDB1 on cell cycle in multiple myeloma
