# Supplementary material for: Increased Expression of SETDB1 Predicts Poor Prognosis in Multiple Myeloma
Source: Biomed Res Int. 2022 Mar 23;2022:3307873. doi: 10.1155/2022/3307873 (PMC8967582; doi:10.1155/2022/3307873)
Supplement: Supplementary Materials — Supplemental Table 1: the information of public databases analyzed in this study. Supplemental Figure1: SETDB1 expression profile of myeloma cells in different databases. Supplemental Figure 2: the potential impact of SETDB1 on cell cycle in multiple myeloma [file 3307873.f1.docx]

Supplemental Table 1. The information of public databases analyzed in this study.

| Accession number | Platform | Country | Numbers of samples | Years | age(median) | Gender  (male/female) | Race  (white/others) | Treatment |
| --- | --- | --- | --- | --- | --- | --- | --- | --- |
| GSE39754 | GPL5175 | USA | HC(N=6)  NDMM(N=170) | 2012 | / | / | / | VAD+ASCT |
| GSE5900 | GPL570 | USA | HC(N=22)  MGUS(N=44)  SMM(N=12) | 2006 | / | / | / | / |
| GSE2658 | GPL570 | USA | MM(N=559) | 2005 | / | / | / | TT2: n=351  TT3: n=208 |
| GSE124435 | GPL16686 | Spain | MM(N=24) | 2018 | 60(46-91) | 10/13/NA | / | / |
| GSE31161 | GPL570 | USA | NDMM(N=780)  RMM(N=160) | 2011 | / | / | / | TT2: n=474  TT3: n=466 |
| GSE9782 | GPL96  GPL97 | USA | MM(N=264) | 2007 | 61(27-86) | 159/105 | 229/35 | PS341: n=188  Dex: n=76 |
| GSE24080 | GPL570 | China | MM(N=554) | 2010 | 58(24.8-76.5) | 334/220 | 492/62 | TT2: n=340  TT3: n=214 |
| GSE136337 | GPL27143 | USA | MM(N=426) | 2019 | 59(32.4-75.8) | 261/165 | 377/49 | THAL+ASCT |
| GSE136324 | GPL27143 | USA | MM(N=867) | 2019 | / | / | / | / |
| GSE26863 | GPL570  GPL9128 | USA | MM(N=304)  MM(N=254) | 2011 | / | / | / | / |
| GSE33685 | GPL10152 | Japan | MM(N=67)  MGUS(N=6) | 2011 | / | 34/39 | 0/73 | / |
| CoMMpass | RNA-seq | / | / | / | / | / | / | / |

HC: healthy control, NDMM: newly diagnosed multiple myeloma, VAD: vincristine, adriamycin, and dexamethasone, ASCT: Autologous Stem Cell Transplant, MGUS: monoclonal gammopathy of unknown significance, SMM: smoldering multiple myeloma, RMM:relapsed multiple myeloma, Dex:dexamethasone, THAL: thalidomide


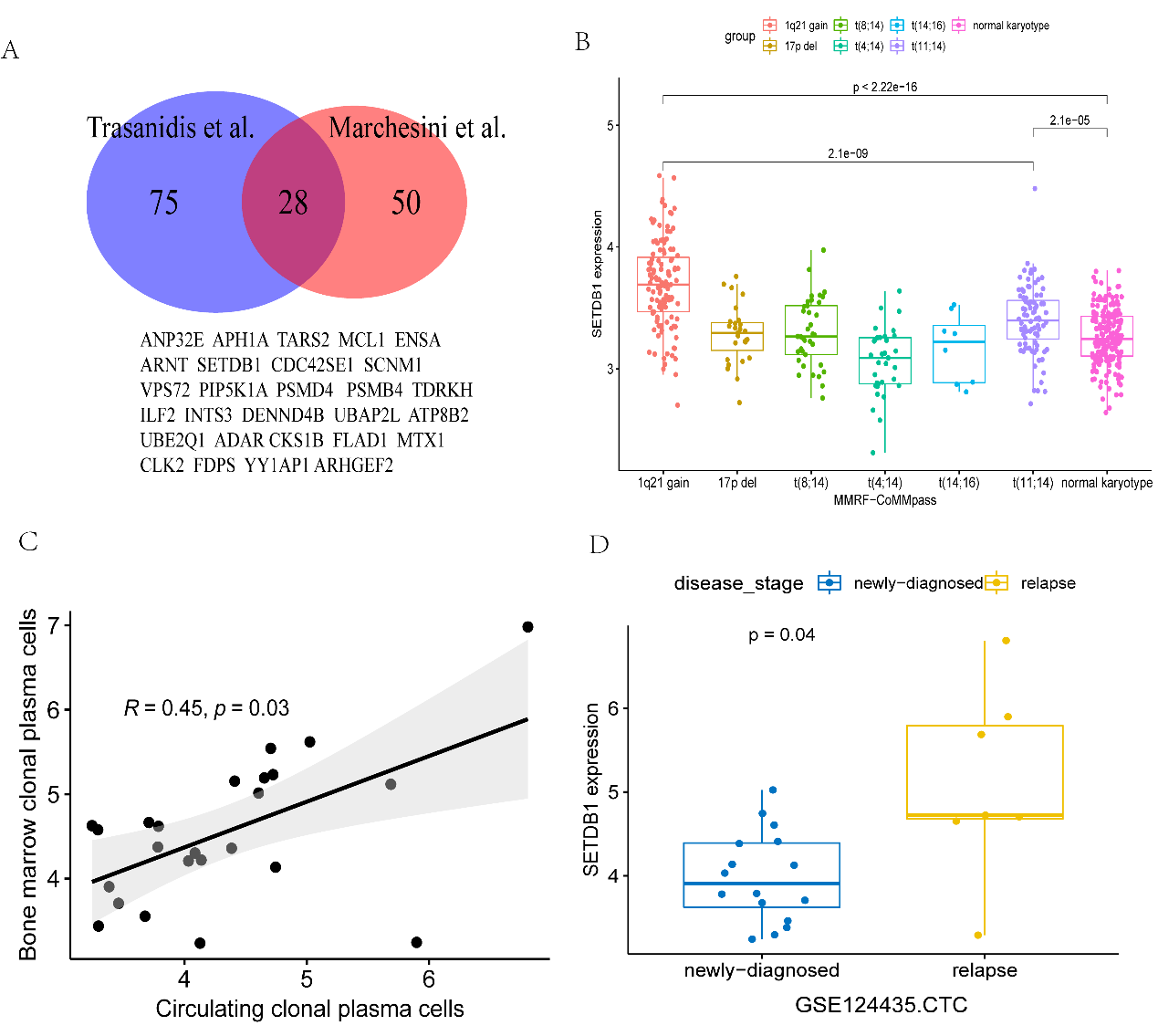


Supplemental Figure1: SETDB1 expression profile of myeloma cells in different databases.

A: Venn-diagram depicting the overlap between genes upregulated in two documents.

B: SETDB1 expression in patients with different cytogenetic abnormalities from MMRF-CoMMpass.

C: The correlation of SETDB1 expression from circulating clonal plasma cells (CTC) and bone marrow clonal plasma cells (BMPC) from GSE124435.

D: SETDB1 expression of CTC was increased in relapsed multiple myeloma compared to NDMM patients from GSE124435.


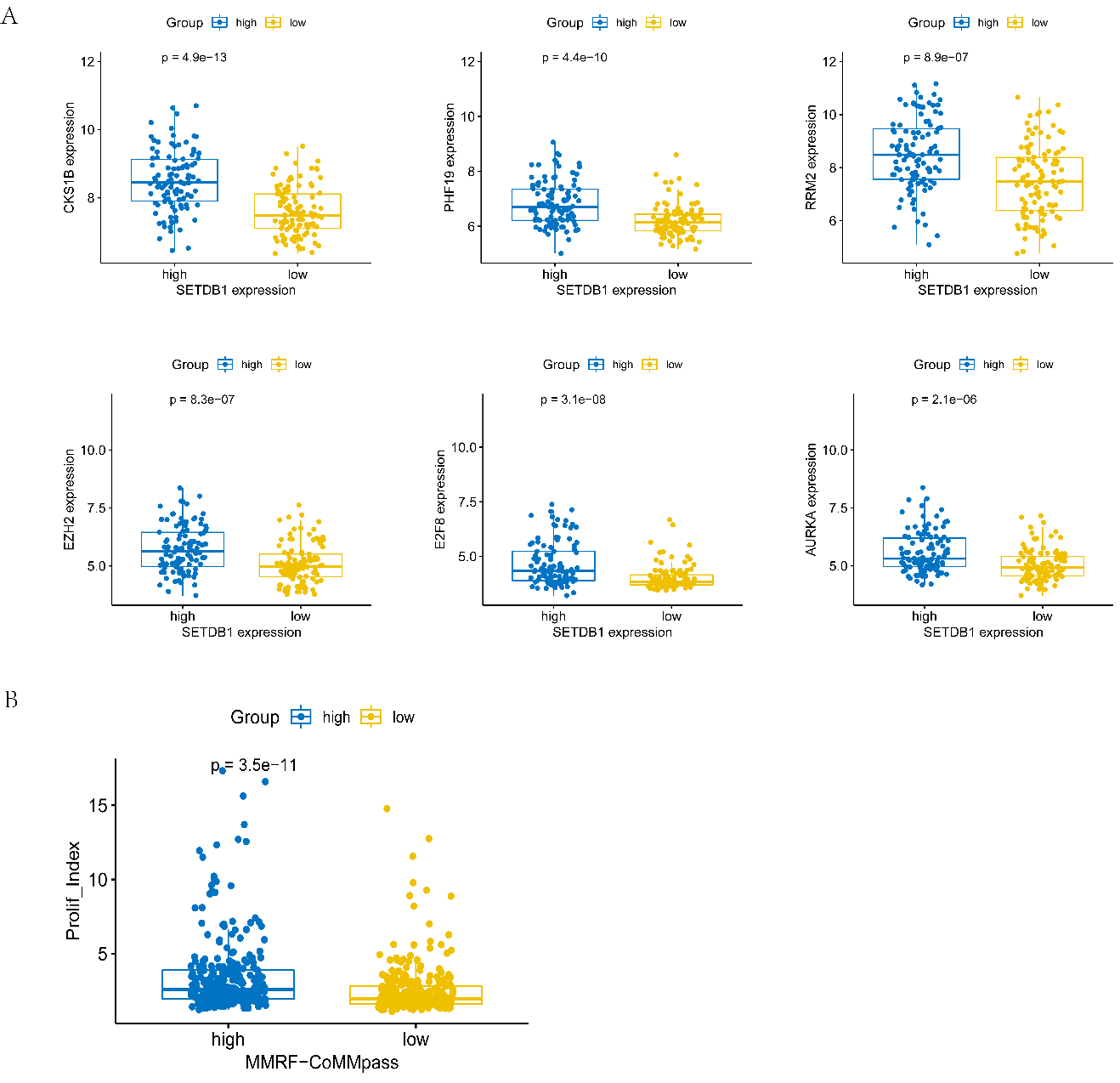


Supplemental Figure2: the potential impact of SETDB1 on cell cycle in multiple myeloma.

A: SETDB1 high expression myeloma cells exhibited increased expression of cell cycle related genes including CKS1B, EZH2, PHF19, E2F8, AURKA, RRM2 compared to low expression group.

B: Proliferation index was significantly increased in SETDB1 high expression patients from MMRF-CoMMMpass.
